# Spatial cover and carbon fluxes of urbanized Sonoran Desert biological soil crusts

**DOI:** 10.1038/s41598-022-09769-7

**Published:** 2022-04-06

**Authors:** Genna Gallas, Mitchell Pavao-Zuckerman

**Affiliations:** 1 Department of Ecology and Evolutionary Biology, University of Arizona, Tucson, AZ USA; 2 Department of Environmental Science and Technology, University of Maryland, 1428 Anim. Sci/Agr. Eng Bldg., College Park, MD 20742-2315 USA

**Keywords:** Environmental sciences, Ecosystem ecology, Microbial ecology, Urban ecology

## Abstract

Biological soil crusts (BSC) are important contributors to nutrient cycling in arid environments such as the Sonoran Desert. BSC at an urban (University Indian Ruins) and at a non-urban site (Santa Rita Experimental Range) were compared to determine if their structure or function was influenced by proximity to an urban environment. The Step Point method was used in the field to determine ground cover; which was found to be similar between sites. However, the spatial distribution of the BSCs was significantly different, such that more BSCs were found under plants at the non-urban site (P < 0.05). Relative gross photosynthesis was measured in the lab by addition of a watering event. Gross photosynthesis was found to be higher in the non-urban BSCs (*P* < 0.001), indicating lowered productivity in urban BSCs due to effects caused by proximity to urban environments. This study provides evidence that BSCs at urbanized sites are affected functionally, and therefore may be contributing differently to carbon and nitrogen cycling in these ecosystems.

## Introduction

Biological soil crusts (BSC) are important primary producers in arid and semiarid ecosystems^1^. They are topsoil communities made up of soil particulates in close association with cyanobacteria and other microbiota that form a coherent layer in the uppermost millimeters of and on top of the soil^2–5^. BSCs cover roughly 12% of the Earth’s land surfaces^6^. Especially in arid and semiarid landscapes, BSCs fill much of the intercanopy space niche unoccupied by plants. Due to their ability to utilize small water pulse events they become the primary producers in the ecosystem^7,8^, where precipitation limits higher plant growth^9^. BSCs have been extensively studied in the Great Plains^10^, the Great Basin Desert^11^, in the Kalahari desert^12^, in the Karoo succulent region of South Africa^6^ and in the Mojave Desert^13^, but in the Sonoran Desert they are less well studied, especially in relation to how they are affected by urbanization. The Sonoran Desert is the hottest desert in the U.S., and the plants that inhabit it are distributed diffusely^14–16^. Sonoran desert BSCs are heavily dominated by a diverse range of cyanobacterial species due to the aridity of the environment^14,17^ . These BSCs can also contain algal, lichen, and bryophyte species^14^. In the Sonoran desert, BSCs are important in stabilizing the soil, allow easier establishment for vascular plants, and are the main contributors to carbon and nitrogen cycling^7,14,18–20^. BSCs are of crucial ecological importance for the survival of higher plant life in this harsh environment. Our study, to our knowledge, is the first to address the gap in knowledge regarding BSCs in the Sonoran desert and their response in terms of structure and function to the urbanization process.

These BSCs that support the desert ecosystem can have their structure and function disrupted in many ways. Structurally, BSCs can be disturbed by mechanical and anthropogenic forces, such as trampling by humans and livestock^14,21^. These disturbances affect the composition of the crust or their ability to maintain interconnectedness. Other physical disturbances are caused by hiking, mining, and off-road vehicles^2^. BSCs are often the first successional organisms to reinhabit an area that has been disturbed^22^, and their cover decreases with less disturbance when chronic disturbance effects are common in a landscape; such as due to grazing by livestock and fires^23^. At times, human disturbance such as roadcutting can actually serve as an ideal habitat of bare soil for crust colonization^10^. These physical disturbances, on the whole, generally reduce crust biodiversity and simplify the composition of the crust to a few or one species of cyanobacteria^14^, and can take decades to millennia to recover^24^.

Other changes may impact the function of BSCs are more indirect in nature, such as those that affect the carbon or nitrogen cycling activity of the BSCs. Anthropogenic nitrogen deposition can decrease or stimulate nitrogen fixation by BSCs, depending on the species composition of the BSCs^25^. For example, BSCs containing *Collema* have been shown to be negatively impacted by waste from coal power plants^26^; while BSCs containing *Nostoc* have increased rates of nitrogen fixation due to short exposure to low concentrations of pollutants^27^. Changes in species composition can impact the nitrogen fixation rates of BSCs, thereby altering ecosystem dynamics by altering nitrogen inputs and outputs^3^. Due to these disruptions, a significant difference between urban and non-urban BSCs is expected. Especially in areas where there are limited vascular plants that fix nitrogen, BSCs that fix nitrogen are critical for nitrogen cycling in those ecosystems^28^. BSCs are known as ecosystem engineers that drive ecological processes that make plant succession after disturbance possible; such as soil development, nutrient uptake, and biogenic weathering^29^. Their importance cannot be understated in arid environments where water is scarce, and they modify soil properties that affect how water moves and infiltrates the soil, and contribute to soil stability^30^.

The process of urbanization influences ecosystem functioning through indirect changes in climate, chemistry, hydrology, and also through direct alteration of community composition and trophic structures^31^. Plant and animal communities alike are homogenized by urbanization^32^. Soil temperatures are influenced by the urban heat island effect, and have been associated with enhanced rates of biogeochemical processes^33–35^. Urban areas generate a dome of carbon dioxide and produce widespread environmental contamination, including heavy metals and aerosols^33^. The amount of nitrogen and carbon found in the atmosphere is higher near urban areas due to increased deposition by the urban environment^33^. Dust clouds and sandstorm events can transport BSC inoculants across the globe^36,37^. The organization of the crust community is typically simplified by many disturbances that do not completely destroy the BSCs’ structure^25^. This results often in a decrease in the diversity of the BSC community. Urban settings also reduce the activity and microbial functioning of BSCs by altering the inputs of organic matter^38^. However, the most profound change to the community of microbes found in BSCs was caused by alteration of the vegetation cover and deviations in how the land is used^38^.This is due mainly to fluctuations that increase the input of nitrogen and carbon into the soil, changing their cycling patterns^38^. It has been shown that in the city core of Phoenix, AZ, more nitrogen is deposited from air pollution in urban areas compared to the surrounding non-urban areas; and previous studies have shown that moss-dominated BSCs are able to take up this excess nitrogen^39^. Changes to the nitrogen cycling in urban areas is expected to affect the activity of the crust community, because BSCs provide important nutrients for higher plants and fix nitrogen in the soil as a main function^47^.

This study is significant in that it adds to the body of knowledge on BSCs. It seeks to answer the question of how urbanization affects the function and structure of BSCs in the Sonoran Desert. To examine this, we compared BSCs from urban and non-urban sites in the laboratory and in the field. The field experiments measured the cover of BSCs and the association between vegetation cover and BSCs in both an urban and non-urban site. In the laboratory, carbon uptake and efflux rates from BSCs were tracked over time. In addition, a water-pulse addition experiment was utilized to determine differences in crust functional responses to precipitation inputs. This study suggests that crusts at urbanized sites are contributing differently to carbon and nitrogen cycling in these ecosystems.

## Materials and methods

### Sites

Biological crust samples and corresponding field data were collected from two sites: an urban and a non-urban site. The urban site is located at University Indian Ruins, Tucson, AZ (Latitude/Longitude: 32.25, −110.84). The site consists of an archeological site owned by the University of Arizona that is notable for being located inside the urban epicenter of Tucson, AZ. This site still maintains a patch of remnant desert ecosystem and thus provides an important BSC source that is urbanized. The non-urban site is located in the Santa Rita Experimental Range (Latitude/Longitude: 31.83336488792502, −110.85155561663484). This site consists of an ecological study site about 40 miles south of Tucson, AZ, far from urban epicenters and removed from the impacts of urbanization. Both sites were similar in climate and ecologically, and were interspersed with bare ground and bunch grasses and were dominated by the creosote bush (*Larrea tridentata)*.

### Percent cover

Percent cover was determined at each site using the Step-Point method of sampling described by Evans and Love^41^. With the step-point method, cover information is collected along a transect, delineated by choosing a landmark on the horizon and walking a straight line in that direction^41^. Every 2.5 m, a point on the ground is determined from the tip of one’s boot, using a straight edge. Data taken at each point consists of what is covering the ground at that point^41^. For this study, three transects separated by 20 m were walked at each site; each transect consisted of the first 100 points encountered (spaced at 2.5 m)^41^.

BSCs were identified at two sites and categorized into several categories: light cyanobacterial BSCs, dark cyanobacterial BSCs, BSCs with lichen, and BSCs with moss. In the Sonoran Desert, typical BSCs consist of microbiota, most importantly including cyanobacteria. In some cases, mosses and lichen are also present in the BSCs. In this study, the crusts were found to be dominated by light and dark cyanobacterial crusts. Moss and lichen species were not identified further. Cover was classified as one of the following categories: bare soil, light cyanobacterial crust, dark cyanobacterial crust, rock, litter, or vegetation. Canopy and foliar cover data was taken according to methods described by^42^. Cover was described in levels, with the first level being the “basal” or ground level- i.e. what is found directly on the ground (level zero). Above the basal level of what was directly on the ground surface, the type of vegetation that was above this was considered to be “Level 1”. If needed, additional levels were added if there was vegetation present above the first level^42^. Specific species were not recorded in this particular case because our goal was to merely determine percent cover. Instead, vegetation was categorized by type: i.e., shrub, grass, etc. This method was used at the sites in order to calculate how much vegetation cover was present^42^.

### Soil biological crust activity: lab assay

To assess biological crust physiological activity, a laboratory study was conducted on field-collected crust and soil samples. Water was added to simulate a precipitation pulse (20 mLs), and respiration and net photosynthesis were tracked over time.

BSCs and 5 cm of soil beneath them were collected from each site. Crust samples were collected from each site at random locations within the same transects that were used to determine percent cover. Crust samples (roughly 6 cm in diameter) were gently lifted from the soil surface with a trowel. Soil from beneath the BSCs was harvested to a depth of 5 cm, and placed in half-pint canning jars. BSCs were carefully placed into jars on top of their ‘home’ soils. These BSCs were stored outside and uncovered, but sheltered from rainfall, to avoid any detrimental effects from artificial lights and temperatures. BSCs were identified using the crust typification used by Bowker et al.^18^, categorizing BSCs as either dark or light cyanobacterial, lichen, or moss. No distinctly lichen or moss crusts were found, thus the crusts studied in these experiments were classified as either light or dark cyanobacterial crusts.

At the location where a crust was sampled, a corresponding soil sample was also taken to determine soil properties at each crust site. The soil samples taken were 5 cm in diameter and 5 cm deep. The soil property samples were dried via air and then sieved through a 4 mm mesh to eliminate rocks and larger debris. Soil organic matter was determined by loss on ignition with a Type 30400 Thermolyne Muffle furnace^43^. pH was determined with a 1:1 volume ratio of soil to diH2O using VWR sympHony pH meter (VWR, Batavia, IL). The mass lost due to combustion was then calculated.

### Soil crust activity: lab incubations

Large rainfall events were simulated by adding 20 mL of water to each crust sample. Preliminary studies on BSCs showed little differences between small (125 mL over a 625 cm^2^ plot) and a medium (312 mL over a 625 cm^2^ plot) simulated pulse events, therefore the large pulse was predicted to show a larger response in terms of CO_2_ flux (data not shown). Paired light and dark measurements of CO_2_ flux from the crusts were determined using a LiCor gas analyzer (LI-7000 CO_2_/H_2_O Analyzer, Lincoln, NB). Paired light and dark measurements were recorded every hour after the water addition for 6 h. Light (photosynthesizing) measurements were taken first, and then after 15 min of acclimating to dark conditions, the dark measurements were taken. Acclimating to the dark also included covering the jars; therefore, the BSCs were receiving a reduced flow of air during this period. Slopes of CO_2_ concentration versus time are used in the calculation of soil flux, adjusted for jar and chamber volume. For quality control purposes, data was checked to ensure that slopes were based on a linear regression of the carbon flux data vs. time and had an r^2^ value of 0.95 or higher. The change in CO_2_ flux between light and dark conditions is assumed to be primarily due to photosynthetic activity.

### Calculations and statistics

Ground Cover, Canopy/Foliar cover, and Basal Cover was calculated using methodology described by Colloudon et al.^42^. Ground cover is defined as the type of material found at the ground surface, i.e., rock, bare soil, plant detritus, etc. ANOVA analysis was used to look at the difference in ground cover between urban and non-urban BSCs. A regression of time vs. flux was done on each urban and non-urban set of data points, and one was done on each dark/light combination. The soil properties were compared using a t-test of both pH and organic matter. No significant differences in soil properties were found between sites (Table 2).

Gross photosynthesis is the total amount of light energy that is converted to biochemical energy, found by subtracting the light treatment flux (photosynthesis) by the dark treatment (respiration). To find its relative value, the gross photosynthesis value was then normalized against the first measurement at time zero, the pre-water treatment measurement. The gross photosynthesis measurements were normalized to the maximum by subtracting the minimum value from each average value, and dividing this result by the maximum minus the minimum. Dark and light measurements of CO_2_ flux were used to determine photosynthetic activity of both urban and non-urban samples.

## Results

### Percent cover

We compared ground cover and cover of BSCs in an urban and non-urban site in the Sonoran Desert. The ground cover mean values did not differ significantly for any of the categories (Table 1), suggesting that these two sites are similar in composition and layout. There is no significant difference (*P* > 0.05) in the amount of ground cover of BSCs in both sites, however, the location where the BSCs were found did differ significantly between sites when analyzed using a two-way ANOVA test (Table 2). There were significantly more BSCs under plants than in the open (*P* < 0.05), and the interaction of site and cover was also significant (*P* < 0.01). Therefore, at the non-urban site there were significantly more BSCs under plants than at the urban site.

**Table 1:** (A) Mean ground cover (%) at urban and non-urban sites in and around Tucson, AZ per transect.

	Non-urban			Urban		
	Mean	SEM	Percent of total	Mean	SEM	Percent of total
**(A): Mean ground cover: difference by site**						
Light cyanobacterial crusts	5.6	0.5	7.5%	3.8	0.3	5.3
Dark cyanobacterial crusts	2.4	0.5	2.7%	3.8	0.4	4.9
Rock	6.4	0.6	8.7%	7.3	0.5	10.3
Litter	25.1	0.8	35.0%	24.4	0.9	34.6
Vegetation/canopy cover	42.1	0.2	40.3%	48.7	0.7	45.0
Total crust cover (combined)	–	–	11.4%	–	–	10.6
% crusts found in open*	–	–	22.4%	–	–	55.8
% crusts found under plants*	–	–	77.7%	–	–	44.3
**(B): Soil properties: difference by site**						
pH	8.54	0.02	–	8.57	0.01	–
Soil organic matter (grams)	0.59	0.12	–	0.43	0.02	–

An asterisk (*) indicates a significant difference found between sites. Statistics were done using a single factor ANOVA test. (B) Soil properties at urban and non-urban sites. Statistics were done using a single factor ANOVA test and no significant differences were found.

**Table 2:** The degrees of freedom, F value, and statistical significance from a single factor ANOVA test determining differences between ground cover percentages, total crust cover, and soil properties at urban and non-urban sites.

Factors	*df*	*F*
**Ground cover percentage**		
Light cyanobacterial crusts	1.5	n.s
Dark cyanobacterial crusts	1.5	n.s
Rock	1.5	n.s
Litter	1.5	n.s
Vegetation/canopy cover	1.5	n.s
**Soil properties**		
pH	1	n.s
Soil organic matter (%)	1	n.s
**Total crust cover**		
Site	1.11	n.s
Open vs. under plant (cover)	1.11	5.85055*
Interaction between site and cover	1.11	12.83538**
**Carbon flux**		
Time	5.69	28.547***
Cyanobacterial type	1.69	n.s
Site	1.69	n.s
Interaction between cyanobacterial type and time	5.69	n.s
Interaction between cyanobacterial type and site	1.69	6.135*
Interaction between site and time	5.60	n.s
Cyanobacterial type * site * time	5.69	n.s
**Gross photosynthesis**		
Time	5.36	n.s
Site	1.36	12.333**
Interaction between site and time	5.36	n.s

Statistical differences between factors for carbon flux and gross photosynthetic rates were determined using a general linear model, and the df, F, and statistical differences are shown below.

* P < 0.05, ** P < 0.01, *** P < 0.001, *n.s.* not significant.

### Soil crust activity: crust incubation and lab assays

#### Carbon flux

**Figure 1:**
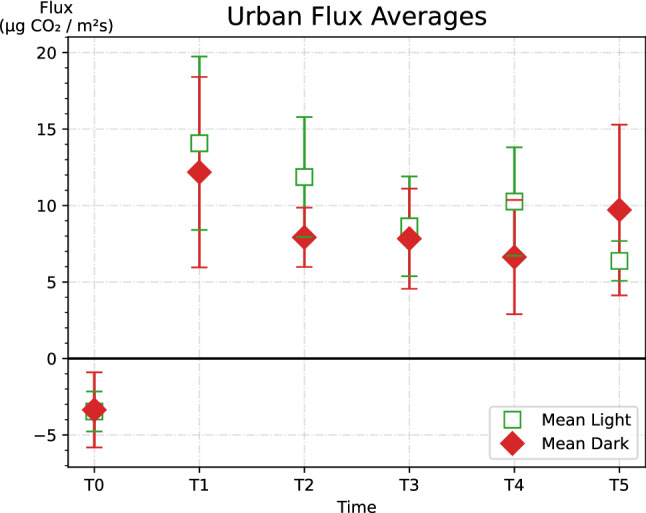
Urban flux averages by dark or light cyanobacterial crust. Error bars represent standard error of the mean.

**Figure 2:**
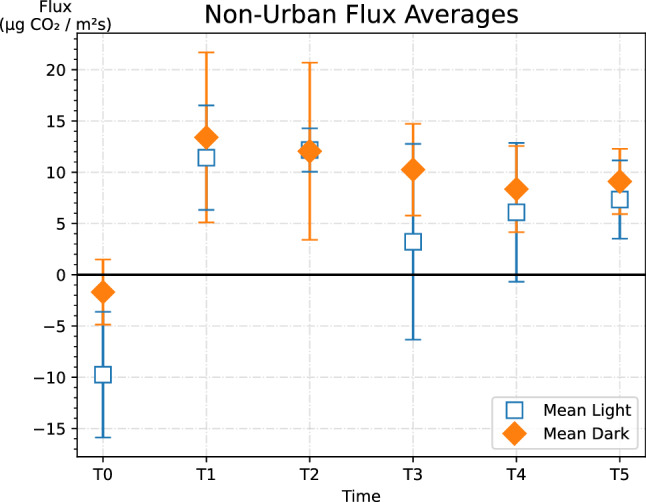
Non-urban flux averages by dark or light cyanobacterial crust. Error bars represent standard error of the mean.

Before watering (t = 0), CO_2_ flux was very low. The rate of CO_2_ emission peaked at 1 h post-watering and slowly declined over the next 6 h. Both light and dark non-urban samples showed negative trends over time after the initial plateau (Fig. 1). Urban samples had negative trends over time as well after their initial plateau (Fig. 2). For the non-urban samples, dark treatments had higher absolute fluxes than light treatments. The urban samples’ light treatments had higher fluxes than dark treatments (Fig. 2). There is little difference in flux between urban and non-urban samples, regardless of whether it was a light or dark treatment. The error bars indicate a substantial variance between samples, suggesting that flux may be more dependent on crust composition than on site. Indeed, the interaction between cyanobacterial type and site was found to be significant using a two-way ANOVA test (P < 0.05) (Table 2). The relationship of flux and site is further explored by looking at relative gross photosynthesis.

### Gross photosynthesis 

**Figure 3:**
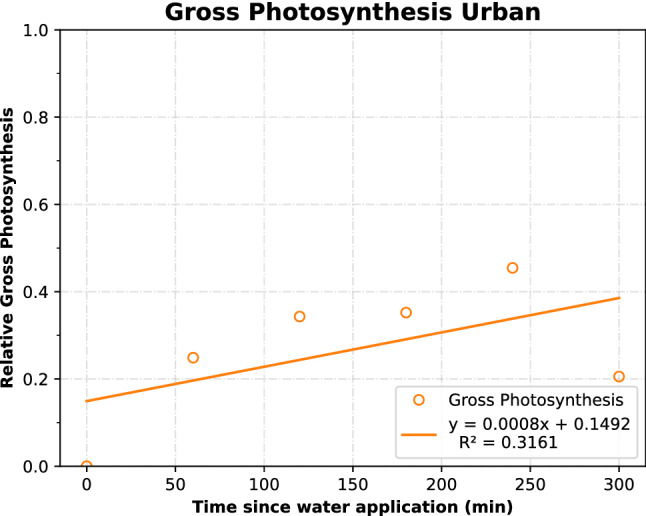
Urban gross photosynthetic rate over time, normalized to the maximum rate.

**Figure 4:**
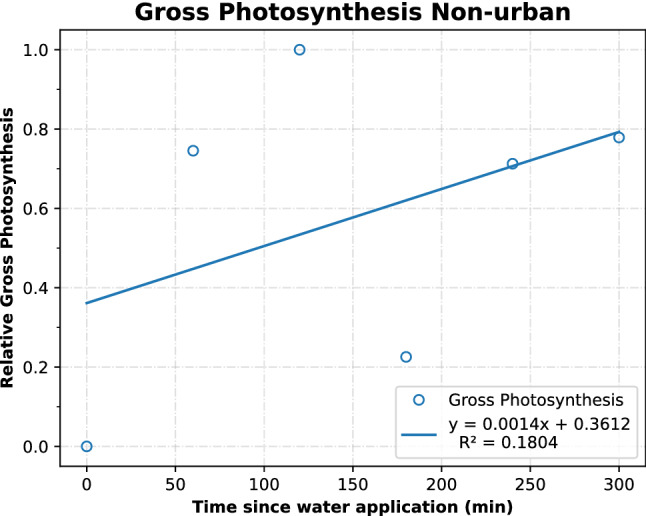
Non-urban gross photosynthetic rate over time, normalized to the maximum rate.

Positive trends are seen for both urban and non-urban samples for normalized gross photosynthesis (Figs. 3 and 4). The non-urban crusts reacted with a greater response of relative gross photosynthesis to the water treatment than the urban BSCs (*P* < 0.01) (Figs. 3 and 4). The linear regressions of the non-urban BSCs showed a higher slope of 1.0 × 10^–3^, while the urban BSCs had a slope of 6.0 × 10^–4^. This indicates that these BSCs increased their gross photosynthesis at a larger rate. However, this was coupled with a less precise fit to the regression for non-urban BSCs.

## Discussion

The mean ground cover of BSCs, rocks, litter, and canopy cover did not vary significantly between urban and non-urban sites. While Ball and Guevara^39^ found less soil crust cover at city core centers of Phoenix, AZ; in our study, the overall percent covered by BSCs did not differ between urban and non-urban sites. This may be due to the fact that remnant urban area in our study had less disturbance to the crusts. However, the proportion of BSCs found under vegetation was significantly altered between sites (P < 0.05). Non-urban BSCs were more often found under plant cover than their urban counterparts. This supports our hypothesis that urban environments will have an effect on the spatial distribution of BSCs. The proximity to urban environments is changing where the BSCs are located, and more of them are located in the open areas, in between vegetation, rather than under vegetation. This may be due to the increased nitrogen deposition in urban environments^33,38^, or increased mixing and porosity of soils due to differences in microclimate or degree of trampling between sites^44,45^. Another hypothesis is that BSCs are more often found underneath plant cover due to the increased moisture retention under vegetation cover. The removal of BSCs by Chamizo et al. and colleagues resulted in a decrease in water infiltration in the soil, causing a decrease in function of BSCs in nutrient cycling resulting in a cascade of downstream effects, including influencing viable vascular plant habitat in arid ecosystems^46^. It has been shown that moisture content is associated with grass biomass and the associated shrub canopy covering it in the Chihuahuan desert^47^. This retention of moisture, however, has also been shown to be caused by the BSCs themselves (depending on parental soil type), producing extracellular polysaccharides that are capable of absorbing moisture^48^. The findings of Berdugo et al. suggest that the presence of BSCs enhances water gain and slowed drying compared to bare soils^49^. This transition of BSCs from under canopy to in the open can have a profound effect on the ecological services the BSCs are providing such as water infiltration, nutrient cycling, and soil stability in urban environments^14,25,46,50^.

We found that for carbon flux there was a significant difference in the interaction between cyanobacterial type and site (P < 0.05). The urban BSCs had a higher percentage of dark cyanobacterial crusts. This may be indicating that there is a shift in the structure and organization, of BSCs depending on the site. While the findings were not statistically significant, a greater proportion (4.85% for urban vs. 2.73% for non-urban) of dark cyanobacterial BSCs were found at the urban site. Proximity to urban areas may affect BSCs through changes in irradiance^51^. This is supported by the findings of Kaya et al.^52^, who found that the urban heat islands in the Istanbul area had a clear association with increased radiance and surface temperatures of the landscape. BSCs protect themselves from the stresses caused by excess radiance through desiccation^53^, reflective fungal covering^54^, and by producing pigments that protect the BSCs from ultraviolet rays^55^. Bowker et al.^18^ describes dark cyanobacterial BSCs as having dark sunscreen pigments to protect the BSCs from high levels of radiance, and the light BSCs may not always form cohesive BSCs the way dark colored BSCs do. This suggests that dark BSCs are more dense and aggregate to the soil better, even though both BSCs are characterized by the same dominant taxa, *Microcoleus vaginatus*^18^. There may be more BSCs under plants at the non-urban site because the BSCs do not have dark sunscreen pigments, thus they rely on the shade of plants instead. In cyanobacterial BSCs such as the ones studied here, scytonemin is the primary UV-protective pigment produced, and this yellow to brown pigment can be up to 15% of the dry biomass of the cyanobacteria^14,55^. These pigments tend to be concentrated in light-exposed tissue (giving a darker color) than in shaded parts of the BSCs; additionally, more heavily pigmented species of cyanobacteria are found nearer the surface layers than less pigmented species^56^. The urban BSCs had a higher percentage of dark cyanobacterial BSCs that may need the dark pigments to act as sunscreen. This suggests that the proximity to urban areas increases the amount of radiance that the BSCs are exposed to.

Gross photosynthesis increased over time following a water application (P < 0.001) and these findings were consistent with previous results from Cable and Huxman^7^. Relative gross photosynthesis was also affected by the site where the BSCs were found, and was higher at the non-urban site than at the urban site (P < 0.01). This suggests that the non-urban BSCs have a greater capacity to respond to water pulses, perhaps due to changed crust structure or impaired functioning because of urban environmental impacts on soils^34,38,39,45,55–59^. Urban crust gross photosynthesis was lowered. This further supports our hypothesis that soil crust functioning is altered by urban environments. This may be caused by pollutants, trampling, or altered crust structure at the urban site^24^. Changes in land use and land cover related to the urbanization of desert landscapes can have consequences for soil properties and microbial community function^38^. For example, areas near Phoenix, AZ had elevated levels of nitrogen, different rates of nutrient cycling, different soil organic matter pools (influencing denitrification), and homogenization of microbial spatial processes^38,39^. Because of their ability to utilize small water pulse events, BSCs are important in fixing much of the carbon and nitrogen in their ecosystem. Whereas higher plants are unevenly spaced, and are unable to grow during drier times, leaving much of the net primary productivity to the BSCs in these carbon-limited soils^16,39,60,61^ Therefore the reduced gross photosynthesis of the urban BSCs compared to non-urban BSCs could have adverse implications for the carbon cycling in urban environments, and many more implications for the ecosystem functions than their abundance would suggest.

This study indicates that BSCs are indeed affected by urban environments by causing changes in their function and structure. In non-urban sites, more BSCs are found underneath the cover of plants, while in urban environments more are found in the open. This has far-reaching implications due to the importance of BSCs for water infiltration, carbon and nitrogen cycling, and many other essential biogeochemical processes. The gross photosynthetic functioning of BSCs was reduced by proximity to urban environments. While both the urban and non-urban BSCs had a similar response over time to the water pulse treatment, the non-urban BSCs’ relative gross photosynthesis was higher than that of the urban BSCs’. This may have been caused by a number of interacting factors, perhaps to the detriment of the urban BSCs. Due to the already proven importance of BSCs in environments where small water pulse events are the main drivers of primary production, the decreased function of urbane BSCs has far-reaching implications as these are important nutrient cyclers in the ecosystem^40^. Further data collection and analysis is needed to tease apart the complex relationship between the BSCs and their environment. Physical disturbances, nitrogen content, heavy metal content, and water infiltration depth into the soil are just some of the factors not yet explored by this study. This study provides useful information about the altered structure and function of BSCs due to urban influence that will be important in ecological management to preserve the essential functions that BSCs provide in the future.

## Data Availability

The datasets generated during and/or analyzed during the current study are available from the corresponding author on reasonable request.
